# Suicide Deaths Among Adolescent and Young Adult Patients With Cancer

**DOI:** 10.1001/jamanetworkopen.2024.42964

**Published:** 2024-11-04

**Authors:** Koji Matsuo, Christina J. Duval, Briana A. Nanton, Jennifer A. Yao, Erin Yu, Christian Pino, Jason D. Wright

**Affiliations:** 1 Division of Gynecologic Oncology, Department of Obstetrics and Gynecology, University of Southern California, Los Angeles; 2 Norris Comprehensive Cancer Center, University of Southern California, Los Angeles; 3 Department of Obstetrics and Gynecology, Los Angeles General Medical Center, Los Angeles, California; 4 Department of Psychology, St Louis University, St Louis, Missouri; 5 Keck School of Medicine, University of Southern California, Los Angeles; 6 Division of Gynecologic Oncology, Department of Obstetrics and Gynecology, Columbia University College of Physicians and Surgeons, New York, New York

## Abstract

This cohort study evaluates differences in suicide death rates across age groups of adolescent and young adult patients with cancer over time in the US.

## Introduction

The overall cancer incidence among adolescent and young adult (AYA) patients is increasing at an alarming rate in the US largely driven by thyroid cancer.^1^ Although cancer mortality continues to decrease among AYA patients,^1^ those who survive cancer are at elevated risk for emotional distress, mental health problems, and suicide.^2^ A prior investigation has demonstrated that the number of suicide-related deaths increased among AYA patients with cancer from 1973 to 2015.^3^ Given the increasing rate of mental health problems, more contemporaneous data for AYA patients with cancer are urgently needed. This study evaluates differences in suicide rates across age groups of patients with cancer over time in the US.

## Methods

Details of this cohort study are described in the eMethods and eTable in Supplement 1. Briefly, this cohort study queried the National Cancer Institute (NCI) Surveillance, Epidemiology, and End Results (SEER) Program^4^ to assess suicide deaths among 4 475 284 deaths from 2000 to 2021. Temporal trends of suicide death rates among deceased patients were assessed according to age and sex. The ethical committee of the University of Southern California exempted institutional review board approval because the NCI SEER Program provided and is the source of the deidentified data used; race and ethnicity, which followed identification provided by the NCI SEER database, were grouped by the program, and the program has not verified and is not responsible for the statistical validity of the data analysis or the conclusions derived by the study team. This study followed the STROBE reporting guideline, and IBM SPSS, version 29.0 was used for statistical analysis. A 2-sided *P* < .05 was defined as statically significant.

## Results

At the cohort level, the median age at cancer diagnosis was 72 years (IQR, 62-80 years). Most patients were non-Hispanic White (3 323 503 of 4 475 284 [74.3%]). More deaths were recorded for male patients compared with female patients (54.3% vs 45.7%). Among 82 malignant neoplasms, the lung and bronchus were the most frequent sites reported (880 748 of 4 475 284 [19.7%]).

At the cohort level, 11 902 suicide deaths were reported (2.7 per 1000 deaths or 1 in 376 deaths). The suicide death rate was highest in the AYA (aged 15-39 years) male patient group among 6 sex- or age-stratified groups (9.0 per 1000 deaths), followed by the adult (aged 40-59 years) male patient group (5.7 per 1000), the AYA female patient group (4.5 per 1000), the older adult (aged ≥60 years) male patient group (3.7 per 1000), the adult female patient group (2.4 per 1000), and the older adult female patient group (0.5 per 1000) (*P* < .001).

Suicide death rates increased in all 6 groups (Figure). However, the AYA male patient group exhibited the highest suicide death rate throughout the study period from 4.9 to 15.4 per 1000 deaths in 2000 and 2021 (*P* < .001 for trend). The gap between the AYA male patient group and the other patient groups widened over time (4.9 vs 0.4-3.1 per 1000 deaths in 2000 and 15.4 vs 0.6-7.4 per 1000 deaths in 2021) (Figure). Among those who had suicide deaths, the median time from cancer diagnosis to suicide deaths in the AYA groups (71 and 67 months for the AYA female and AYA male patient groups, respectively) was longer than the other age groups (range, 30-54 months; *P* < .001).

**Figure.**
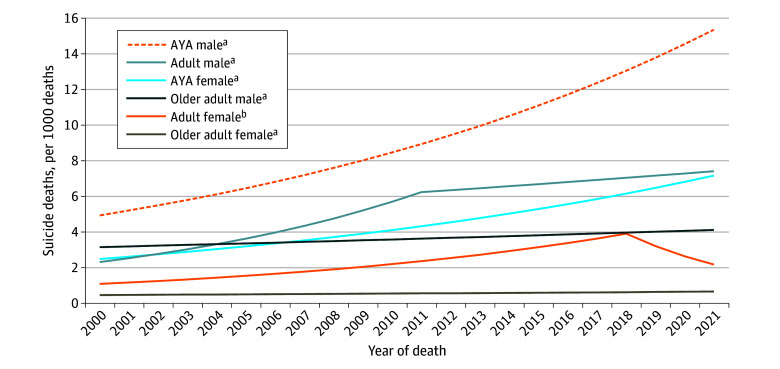
Temporal Trends Associated With Suicide Deaths Among Patients With Cancer per Age and Sex Adolescent and young adult (AYA) was defined as 15 to 39 years; adult, 40 to 59 years; and older adult, 60 years or older. Modeled values are shown from 2000 to 2021: 4.9 to 15.4 per 1000 deaths for the AYA male patient group (*P* = .003 for trend); 2.3 to 7.4 for the adult male patient group (*P* < .001 for trend); 2.5 to 7.2 for the AYA female patient group (*P* < .001 for trend); 3.1 to 4.1 for the older adult male patient group (*P* = .001 for trend); 1.1 to 3.9 from 2000 to 2018 for the adult female patient group (*P* < .001 for trend), followed by a nonsignificant trend from 2018 to 2021 (*P* = .10 for trend); and 0.4 to 0.6 for the older adult female patient group (*P* = .04 for trend). When excluding deaths due to noncancer reasons, increasing temporal trends were consistent in all the groups, including the AYA male patient group (5.5-19.9 per 1000 deaths) and the AYA female patient group (2.5-8.6 per 1000 deaths). ^a^*P* < .05 for trend. ^b^*P* < .001 for trend (2000-2018).

A total of 456 sex- or age-stratified types of malignant neoplasms were assessed for suicide deaths among deceased patients. The proportion of suicide deaths exceeded 1% in nearly 30% of reported types malignant neoplasms in the AYA male patient group (22 of 74 [29.7%]), followed by the adult male patient group (12 of 62 [16.2%]) and the AYA female patient group (11 of 78 [14.1%]). When further evaluated for specific type of malignant neoplasm (Table), there were 5 types in which the proportion of suicide deaths exceeded 2%, of which 4 of 5 (80%) were in AYA patient groups: AYA female patients with thyroid cancer (38.7 per 1000 deaths), AYA male patients with thyroid cancer (36.6 per 1000 deaths), AYA male patients with testicular cancer (36.3 per 1000 deaths), and AYA male patients with cutaneous melanoma (24.4 per 1000 deaths).

**Table.** Suicide Death Rates per Sex- or Age-Stratified Types of Malignant Neoplasms

Rank	Sex	Age	Site of malignant neoplasm^a^	No.	Suicide rate per 1000 deaths (SE)	Median (IQR) time to suicide death, mo^b^
1	Female	AYA	Thyroid	826	38.7 (6.7)	101 (40-131)
2	Male	AYA	Thyroid	465	36.6 (8.7)	101 (82-154)
3	Male	AYA	Testis	2313	36.3 (3.9)	64 (33-122)
4	Male	AYA	Melanoma of the skin	1805	24.4 (3.6)	78 (38-138)
5	Male	Adult	Testis	1663	22.2 (3.6)	86 (31-168)
6	Male	Adult	Lip	647	18.5 (5.3)	62 (43-93)
7	Male	AYA	B-cell neoplasms	814	17.2 (4.6)	71 (45-103)
8	Male	Adult	Pharynx and oral cavity other	651	16.9 (5.1)	41 (7-60)
9	Male	Adult	Melanoma of the skin	12 188	16.0 (1.1)	73 (32-120)
10	Male	AYA	Hodgkin lymphomas	1198	15.0 (3.5)	63 (18-119)
11	Male	Adult	Prostate	39 295	14.7 (0.6)	67 (32-120)
12	Male	Adult	Thyroid	3003	14.3 (2.2)	51 (9-93)
13	Female	AYA	Melanoma of the skin	1234	12.2 (3.1)	104 (61-149)
14	Male	Adult	Major salivary glands	1207	11.6 (3.1)	41 (8-118)
15	Female	Adult	Thyroid	4569	10.9 (1.5)	74 (35-156)
16	Male	Adult	B-cell neoplasms	4100	9.8 (1.5)	71 (26-132)
17	Male	Adult	Larynx	7655	9.7 (1.1)	30 (16-77)
18	Male	Adult	Floor of mouth	1778	9.6 (2.3)	42 (16-72)
19	Male	Adult	Other hematopoietic neoplasms	3595	9.5 (1.6)	62 (38-92)
20	Male	Adult	Oropharynx	11 384	9.3 (0.9)	52 (13-92)
21	Male	AYA	Kidney parenchyma	1193	9.2 (2.8)	57 (21-84)
22	Male	Adult	B-cell neoplasms	7403	9.1 (1.1)	55 (15-111)
23	Male	Older adult	Tongue anterior	6188	9.0 (1.2)	19 (6-47)
24	Male	Adult	Tongue anterior	2943	8.8 (1.7)	34 (10-68)
25	Male	Older adult	Oropharynx	19 491	8.8 (0.7)	15 (4-41)
26	Female	Adult	Melanoma of the skin	6164	8.6 (1.2)	70 (43-123)
27	Male	Adult	Urinary bladder	12 524	8.5 (0.8)	59 (23-106)
28	Male	Adult	Hodgkin lymphomas	2006	8.5 (2.0)	39 (17-83)
29	Male	Adult	Small intestine	2511	7.6 (1.7)	54 (12-100)
30	Male	Adult	Anus, anal canal, and anorectum	2654	7.5 (1.7)	50 (35-86)

Abbreviation: AYA, adolescent and young adult.

^a^ According to the 2023 site recode *International Classification of Disease for Oncology, Third Edition* revision, site was stratified according to sex (female or male) and age at cancer diagnosis (AYA [15-39 years], adult [40-59 years], and older adult [≥60 years]). A total of 456 patient groups were assessed for suicide deaths among deceased patients. Groups with a low number of suicide-related events (1-10) are not shown.

^b^ From diagnosis to suicide death.

## Discussion

Increasing trends of suicide death among AYA patients with cancer observed in this study period extending to the early 2020s add important information because a prior investigation ended in the mid-2010s.^3^ Because sex-specific trends among AYA patients with cancer were not examined previously,^3^ the rapidly increasing suicide death rate among AYA male patients with cancer is alarming. In 2021, 1 in 65 deaths were attributed to suicide in this group. Key limitations in this study included lack of information about mental health conditions, including suicidal ideation, past suicide attempts, malignancy status such as disease recurrence or remission, reason for or method of suicide attempt or death, comparison with general population, and, although rare, use of physician-assisted suicide death.

Together with the population-level increase in the US suicide death rate,^5^ the results of this assessment call for attention focused on the increasing suicide death rate among AYA patients with cancer, particularly male individuals. The proportion of AYA patients with cancer of thyroid, testis, or cutaneous melanoma who had a suicide death was greater than 2%, and they most benefit from a psychosocial and mental health evaluation.^6^ Because this study noted that many suicide deaths among these AYA patients with cancer occur years after the cancer diagnosis, long-term care and support for cancer survivors is recommended.

## Notes

Supplement 1. **eMethods.** Material and Method
eReferences.
**eTable.** Study Population for Malignancy Site

Supplement 2.
Data Sharing Statement
