# Supplementary material for: Suicide Deaths Among Adolescent and Young Adult Patients With Cancer
Source: JAMA Netw Open. 2024 Nov 4;7(11):e2442964. doi: 10.1001/jamanetworkopen.2024.42964 (PMC11536222; doi:10.1001/jamanetworkopen.2024.42964)
Supplement: Supplement 1. — eMethods. Material and Method eReferences. eTable. Study Population for Malignancy Site [file jamanetwopen-e2442964-s001.pdf]

Supplemental Online Content

Matsuo K, Duval CJ, Nanton BA, et al. Suicide deaths among adolescent and young adult patients with cancer. *JAMA Netw Open*. 2024;7(11):e2442964. doi:10.1001/jamanetworkopen.2024.42964

eMethods. Material and Method.

eReferences.

eTable. Study Population for Malignancy Sites

This supplemental material has been provided by the authors to give readers additional information about their work.

© 2024 Matsuo K et al. *JAMA Network Open*.

eMethods. Material and method.

The University of Southern California Institutional Review Boards deemed the study exempt as it included only publicly available, deidentified data (registration number: HS-16-00481). This retrospective cohort study queried the National Cancer Institute's Surveillance, Epidemiology, and End Results program.[1] Their data capturing mechanism represent more than 97% of new incident cancer cases in the registered area, and the most recent version includes approximately 35% of U.S. populatoins.[2]

We selected patients with cancer diagnosed from 2000-2021 who had died and limited the cohort to the 4,475,284 patients with data on cause of death from 8,687,025 patients (eTable). This approach was to assess suicidal deaths in cancer patients from the different aspect to prior population-level investigation.[3]

The exposure was patient age at cancer diagnosis, grouped into adolescents and young adults (AYA) (15-39 years), adult (40-59 years), and older adult (≥60 years). The main outcome was deaths attribute to suicide. The survival data in the program is externally linked to the National Death Index annually.

Suicidal death rates among deceased patients were summarized in each year, and temporal trends assessed according the patient age and sex strata by fitting linear segmented regression models with log-transformation with one-year time increments. The secondary outcomes included cancer type-specific suicidal deaths based on age and sex stratification. Categorization of primary tumor site was based on definitions described by the National Cancer Institute.[4]

Statistic interpretation followed two-tailed hypothesis and a *P*-value of less than .05 was defined as statistical significance. Statistical Package for Social Sciences (IBM SPSS, version 29.0, Armonk, NY, USA) was used for the analysis. This study followed the STROBE reporting guidelines for cohort studies.

eReferences.

1. The Surveillance, Epidemiology, and End Results Program. National Cancer Institute. <<https://seer.cancer.gov/index.html>> (accessed 6/24/2024).

2. Hicks-Courant K, Ko EM, Matsuo K, Melamed A, Nasioudis D, Rauh-Hain JA, Uppal S, Wright JD, Ramirez PT. Secondary databases in gynecologic cancer research Int J Gynecol Cancer 2024:ijgc-2024-005677. doi: 10.1136/ijgc-2024-005677.

3. Yang P, Zhang L, Hou X. Incidence of suicide among adolescent and young adult cancer patients: a population-based study. *Cancer Cell Int.* 2021;21(1):540.

4. Site Recode ICD-O-3 2023 Revision Expanded Definition. Surveillance, Epidemiology, and End Results Program. National Cancer Institute.
https://seer.cancer.gov/siterecode/icdo3_2023_expanded/ (accessed 6/24/2024).

© 2024 Matsuo K et al. *JAMA Network Open*.

eTable. Study population for malignancy site.

Malignancy site [a]	No (%)
Total	4475284 (100)
Lung And Bronchus	880,748 (19.7)
Colon And Rectum (Excluding Appendix)	456,591 (10.2)
Prostate	416,022 (9.3)
Breast	402,841 (9.0)
Pancreas	206,837 (4.6)
Urinary Bladder	203,505 (4.5)
Miscellaneous Neoplasms	136,663 (3.1)
Melanoma Of The Skin	121,850 (2.7)
Kidney Parenchyma	120,831 (2.7)
Liver	112,048 (2.5)
Stomach	102,565 (2.3)
Corpus	90,117 (2.0)
Other B-cell leukemia/lymphomas or Lymphoma, NOS	84,640 (1.9)
Plasma Cell Neoplasms	78,592 (1.8)
Brain (Malignant)	78,491 (1.8)
Large B-cell lymphoma	76,539 (1.7)
Ovary	74,877 (1.7)
Esophagus	68,517 (1.5)
Myelodysplastic Syndromes	62,619 (1.4)
Acute Myeloid Leukemias	60,245 (1.3)
Chronic lymphocytic leukemia (CLL)/Small lymphocytic lymphoma	55,029 (1.2)
Myeloproliferative/Myelodysplastic syndromes, including MDS/MPN overlap	43,982 (1.0)
Oropharynx	39,475 (0.9)
Larynx	38,241 (0.9)
Thyroid	29,490 (0.7)
Soft Tissue	29,156 (0.7)
Cervix	29,062 (0.6)
Small Intestine	21,446 (0.5)
Gallbladder	17,784 (0.4)
Intrahepatic Bile Duct	17,063 (0.4)
Anus, Anal Canal And Anorectum	16,273 (0.4)
Extrahepatic Bile Ducts	16,196 (0.4)
Other Non-Epithelial Skin	16,077 (0.4)
Tongue Anterior	15,771 (0.4)
Mesothelioma	15,410 (0.3)
Other T and NK-cell leukemias/lymphomas	14,794 (0.3)
Vulva	12,598 (0.3)
Retroperitoneum And Peritoneum	12,324 (0.3)
Hodgkin Lymphomas	11,628 (0.3)
Digestive Other	11,260 (0.3)
Kidney Renal Pelvis	11,203 (0.3)
Major Salivary Glands	11,090 (0.2)
Precursor Lymphoid Neoplasms	9,684 (0.2)
Hypopharynx	9,066 (0.2)
Other Leukemias	8,496 (0.2)
Ampulla Of Vater	7,809 (0.2)
Gum	7,203 (0.2)
Ureter	6,951 (0.2)
Floor Of Mouth	6,926 (0.2)
Appendix	6,740 (0.2)
Bones And Joints	6,668 (0.1)
Nasal Cavity And Paranasal Sinuses	6,539 (0.1)
Nasopharynx	6,210 (0.1)
Lip	6,117 (0.1)

© 2024 Matsuo K et al. *JAMA Network Open*.

Eye And Orbit	5,945 (0.1)
Testis	4,771 (0.1)
Vagina	4,620 (0.1)
Kaposi Sarcoma	4,253 (0.1)
Biliary Other	4,101 (0.1)
Fallopian Tube	4,075 (0.1)
Penis	4,056 (0.1)
Buccal Mucosa	3,152 (0.1)
Pharynx And Oral Cavity Other	3,139 (0.1)
Adnexa Other And Genital Female Other	2,909 (0.1)
Thymus	2,856 (0.1)
Mycosis Fungoides/Sezary Syndrome	2,713 (0.1)
Mouth Other	2,479 (0.1)
Adrenal Gland	2,403 (0.1)
Urethra	2,211 (<0.1)
Urinary Other	2,194 (<0.1)
Heart, Mediastinum And Pleura	1,969 (<0.1)
Palate Excluding Soft And Uvula	1,645 (<0.1)
Meninges (Malignant)	1,218 (<0.1)
CNS Other (Malignant)	1,134 (<0.1)
Sinus Other	1,017 (<0.1)
Genital Male Other	974 (<0.1)
Miscellaneous Hematopoietic Neoplasms	931 (<0.1)
Trachea, And Respiratory Other	790 (<0.1)
Intracranial Gland (Malignant)	402 (<0.1)
Parathyroid	226 (<0.1)
Endocrine Other	115 (<0.1)
Placenta	87 (<0.1)
Race / ethnicity	
Non-Hispanic White	3,323,503 (74.3)
Non-Hispanic Black	466,552 (10.4)
Hispanic	395,643 (8.8)
Asian / Pacific Islander	258,107 (5.8)
Native American	26,173 (0.6)
Unknown	5,306 (0.1)

[a] Malignancy site according to the 2023 site recode ICD-O-3 revision are shown.

© 2024 Matsuo K et al. *JAMA Network Open*.
