# Supplementary material for: Suicide Deaths Among Adolescent and Young Adult Patients With Cancer
Source: JAMA Netw Open. 2024 Nov 4;7(11):e2442964. doi: 10.1001/jamanetworkopen.2024.42964 (PMC11536222; doi:10.1001/jamanetworkopen.2024.42964)
Supplement: Supplement 2. — Data Sharing Statement [file jamanetwopen-e2442964-s002.pdf]

Data Sharing Statement

Matsuo. Suicide Deaths Among Adolescent and Young Adult Patients With Cancer. *JAMA Netw Open*. Published November 04, 2024. doi:10.1001/jamanetworkopen.2024.42964

Data
Data available: No

Additional Information
Explanation for why data not available: Data sharing statement: The data on which this study is based are National Cancer Institute's Surveillance, Epidemiology, and End Results Program (https://seer.cancer.gov/).
